# Different Effects of Human Umbilical Cord Mesenchymal Stem Cells on Glioblastoma Stem Cells by Direct Cell Interaction or Via Released Soluble Factors

**DOI:** 10.3389/fncel.2017.00312

**Published:** 2017-10-13

**Authors:** Adriana Bajetto, Alessandra Pattarozzi, Alessandro Corsaro, Federica Barbieri, Antonio Daga, Alessia Bosio, Monica Gatti, Valerio Pisaturo, Rodolfo Sirito, Tullio Florio

**Affiliations:** ^1^Section of Pharmacology, Department of Internal Medicine and Centre of Excellence for Biomedical Research (CEBR), University of Genova, Genova, Italy; ^2^Gene Transfer Lab, IRCCS-AOU San Martino-IST, Genova, Italy; ^3^International Evangelical Hospital, Genova, Italy

**Keywords:** mesenchymal stem cell, cancer stem cell, glioblastoma, chemokine, CXCR2, spheroid, co-culture

## Abstract

Glioblastoma (GBM), the most common primary brain tumor in adults, is an aggressive, fast-growing and highly vascularized tumor, characterized by extensive invasiveness and local recurrence. In GBM and other malignancies, cancer stem cells (CSCs) are believed to drive invasive tumor growth and recurrence, being responsible for radio- and chemo-therapy resistance. Mesenchymal stem cells (MSCs) are multipotent progenitors that exhibit tropism for tumor microenvironment mediated by cytokines, chemokines and growth factors. Initial studies proposed that MSCs might exert inhibitory effects on tumor development, although, to date, contrasting evidence has been provided. Different studies reported either MSC anti-tumor activity or their support to tumor growth. Here, we examined the effects of umbilical cord (UC)-MSCs on *in vitro* GBM-derived CSC growth, by direct cell-to-cell interaction or indirect modulation, via the release of soluble factors. We demonstrate that UC-MSCs and CSCs exhibit reciprocal tropism when co-cultured as 3D spheroids and their direct cell interaction reduces the proliferation of both cell types. Contrasting effects were obtained by UC-MSC released factors: CSCs, cultured in the presence of conditioned medium (CM) collected from UC-MSCs, increased proliferation rate through transient ERK1/2 and Akt phosphorylation/activation. Analysis of the profile of the cytokines released by UC-MSCs in the CM revealed a strong production of molecules involved in inflammation, angiogenesis, cell migration and proliferation, such as IL-8, GRO, ENA-78 and IL-6. Since CXC chemokine receptor 2 (CXCR2), a receptor shared by several of these ligands, is expressed in GBM CSCs, we evaluated its involvement in CSC proliferation induced by UC-MSC-CM. Using the CXCR2 antagonist SB225002, we observed a partial but statistically significant inhibition of CSC proliferation and migration induced by the UC-MSC-released cytokines. Conversely, CXCR2 blockade did not reduce the reciprocal tropism between CSCs and UC-MSCs grown as spheroids. In conclusion, we show that direct (cell-to-cell contact) or indirect (via the release of soluble factors) interactions between GBM CSCs and UC-MSCs in co-culture produce divergent effects on cell growth, invasion and migration, with the former mainly causing an inhibitory response and the latter a stimulatory one, involving a paracrine activation of CXCR2.

## Introduction

Glioblastoma (GBM, astrocytoma grade IV according to the WHO classification) is the most common and aggressive brain tumor, characterized by rapid growth and poor prognosis because of outstanding characteristics of invasiveness and recurrence (Brat et al., 2007; Verhaak et al., 2010). Although in recent years considerable progress has been made in the development of novel treatment approaches for GBM, the median survival time is still less than 15 months (Stupp et al., 2009). The primary treatment option for GBM is multimodal, including surgery, followed by radiation and chemotherapy. However, the infiltrating nature of GBM prevents complete surgical resection and the tumor rapidly relapses. Moreover, the benefits of radio- and chemo-therapy are also limited by the presence of the blood-brain barrier and high toxicity of these treatments.

Small populations of cancer cells, named cancer stem cells (CSCs), play a primary role in the development and recurrence of GBM and most of solid and hematological tumors. CSCs, like normal stem cells, have the capacity to self-renew, which grants their persistence within the tumor mass, and to differentiate into different cell phenotypes originating the main cell populations forming the tumor (Cruceru et al., 2013). The presence of CSCs confers to GBM not only a great degree of phenotypic and cellular heterogeneity, but also the resistance to chemo- and radio-therapy (Singh et al., 2004; Florio and Barbieri, 2012; Tanase et al., 2013; Codrici et al., 2016). When transplanted into immunocompromised mice, CSCs generate tumors that retain the same histological features and cell heterogeneity of the original neoplasia (Singh et al., 2004; Friedmann-Morvinski and Verma, 2014). Moreover, a growing body of evidence supports CSC plasticity and the de-differentiation ability of non-CSC “differentiated” tumor cells into CSCs in response to microenvironmental factors (Friedmann-Morvinski, 2014; Suva et al., 2014).

Therefore, CSCs play a crucial role in the evolution of neoplastic diseases and represent a mandatory target to obtain more efficacious therapeutic responses in tumors (Tanase et al., 2013). Similarly to normal stem cells, CSCs isolated from postsurgical human GBM specimens and cultured in chemically defined medium (without serum and containing EGF and bFGF), grow *in vitro* as spheroids and, typically, although not always, express CD133 surface marker (Ludwig and Kornblum, 2017). Importantly, in these culture conditions, CSCs are able to self-renew and retain tumorigenic activity.

Mesenchymal stem cells (MSCs) are non-hematopoietic progenitor cells, originally identified in bone marrow as mononuclear cells that exhibit the capacity to differentiate into different connective tissue cell types, such as adipocytes, osteocytes and chondrocytes (Jiang et al., 2002). Although bone marrow is the most widely used source of MSCs, these cells can be easily isolated from a variety of tissues including adipose tissue, placenta, umbilical cord (UC), UC blood, dental pulp, periodontal ligament and endometrium (Lv et al., 2014).

In recent years MSCs have gained growing interest for their intrinsic property to home in damaged tissues, inflammatory sites and tumors, as well as for their therapeutic potential as tumor-tropic vectors (Rhee et al., 2015). MSCs possess a marked tropism toward several types of tumors, including melanoma, Kaposi sarcoma, Ewing sarcoma, fibrosarcoma, colon, ovarian, pancreatic, breast and renal carcinomas, and GBM (Bexell et al., 2010). In addition, MSCs exhibit tumor suppressor activity in experimental models of glioma, Kaposi sarcoma, malignant melanoma and other tumors (Rhee et al., 2015; Zhang et al., 2017). MSCs were also reported to support tumor growth and metastasis in different malignancies, including colon cancer, lymphoma and melanoma (Klopp et al., 2011; Yagi and Kitagawa, 2013). Although MSCs are non-tumorigenic when xenotransplanted in immune-deficient animals, they could favor engraftment and progression of cancer cells due to immunosuppressive and pro-angiogenic properties (Melzer et al., 2017; Ridge et al., 2017).

UC is a suitable source of MSCs, alternative to bone marrow. UC-MSCs are plastic-adherent when cultivated *in vitro* and, similarly to MSCs derived from other sources, show CD73, CD90 and CD105 surface markers (Dominici et al., 2006), while they do not express MHC-II antigens (Troyer and Weiss, 2008). UC-MSCs attracted increasing attention due to their large availability, easy collection, fast self-renewal, multipotency, low immunogenicity and the absence of tumorigenicity. Based on their migratory capability towards cancer cells, many reports have proposed MSCs as cell therapy to target tumors and to locally deliver anti-cancer molecules. However, a specific tropism of UC-MSCs toward CSCs has been rarely described (Shinojima et al., 2013; Lee et al., 2014; Liu et al., 2014).

MSC homing and migration toward different sites of activity is mainly mediated by the interactions of chemokines and chemokine receptors. Chemokines are organized as different families of peptides produced and released by normal and neoplastic cells, and are defined on the basis of their ability to direct migration of leukocytes. Chemokines exert their biological function through the binding to a large family of G-protein coupled receptors (Rostène et al., 2011), playing a relevant role in the regulation of GBM CSC survival and proliferation (Würth et al., 2014). In particular, the chemokines IL-8, GROβ and GROα are involved in cell migration and angiogenesis through the binding to a common receptor, named CXC chemokine receptor 2 (CXCR2). CXCR2 is a rather promiscuous receptor since it also binds other chemokines: GROβ, ENA-78, GCP-2 and NAP-2; furthermore, a second receptor, CXCR1, shares with CXCR2 some ligands (IL-8, GCP-2 and NAP-2).

Here, we investigated the effects of UC-MSCs on the growth and migration of CSCs isolated from three different human GBMs, and the bidirectional tropism between these cell populations evaluated *in vitro* by 3D spheroids and monolayer cell co-cultures. We demonstrate that UC-MSCs and CSCs own reciprocal tropism when cultured in 3D, and that their direct interaction in co-culture affects each other growth rate. However, UC-MSC-released soluble factors stimulate CSC proliferation through ERK1/2 and Akt activation. Using pharmacological inhibitors, we show that CXCR2 ligands released by UC-MSC promote CSC growth, possibly representing autocrine/paracrine factors that support GBM CSC proliferation.

Notably, co-cultures of CSCs and UC-MSCs form strong and compact spheroids, unresponsive to CXCR2 inhibition, suggesting that physical contact between GBM-CSCs and UC-MSCs, likely mediated by adhesion molecules, is also an important regulator of their biological behavior in addition to, and independently from the soluble chemotactic molecules released.

## Materials and Methods

### Glioblastoma Stem Cell Isolation and Growth Conditions

Following informed consent and Institutional Ethical Board approval, tumor samples, classified as glioblastoma grade IV (GBM) based on the World Health Organization (WHO) criteria, were obtained from three patients (48, 41 and 67 year-olds, two females, one male) undergoing surgical treatment at the Neurosurgery Department, IRCCS-AOU San Martino-IST (Genova, Italy). Patients underwent surgery for the first time and did not receive chemotherapy or radiotherapy. Tumors were immediately processed for single cell isolation by mechanical dissociation and plated in serum-free medium (complete stem medium) containing: Neurobasal™ medium 50%, Dulbecco’s modified Eagle’s medium/F12 50%, B27 supplement (ThermoFisher Scientific), 2 mM L-glutamine, 100 U/ml penicillin/streptomycin, 2 μg/ml heparin, 15 μg/ml insulin, 20 ng/ml bFGF and 20 ng/ml EGF (PeproTech; Bajetto et al., 2013). Cells were used at *in vitro* culture passages 3–8. To induce cell differentiation, CSC cultures were shifted to growth factor-deprived medium containing 10% fetal bovine serum (FBS, Lonza) for at least 2 weeks.

### Glioblastoma Stem Cell Phenotype Characterization by Immunofluorescence

Cells were fixed with 4% paraformaldehyde, permeabilized with 0.1% Triton X-100 and immunostained with anti-GFAP and anti-SOX2 (Abcam, Cambridge UK) antibodies, followed by fluorochrome-conjugated secondary antibodies (Molecular Probes, OR, USA) and counterstained with DAPI (Sigma-Aldrich, Milano, Italy; Würth et al., 2017). Images were acquired with DM2500 microscope (Leica, Milano, Italy) equipped with DFC350FX digital camera (Leica).

### Isolation and Culture of Human Umbilical Cord-Derived Mesenchymal Stem Cells (UC-MSCs)

Seven human UCs (UC 1–7) of both sexes were collected from women undergoing full-term pregnancy elective cesarean section, at the Gynecology and Obstetrics Department of International Evangelical Hospital (Genova, Italy), after written informed consent and approval by Institutional Ethical Board. Cords were immediately processed and, after washing in PBS and vessel removal, mechanically dissociated and placed in MesenPRO RS™ Medium (Gibco, ThermoFisher Scientific), with 100 U/ml penicillin/streptomycin (Lonza), at 37°C in 5% CO_2_ in air atmosphere. Adherent cells were passed when they reached about 80% confluence. Passages between two and six were used for the experiments (Angeletti et al., 2016).

### Characterization of UC-MSCs by FACS Analysis

UC-MSCs were detached with StemPro^®^ Accutase^®^ Cell Dissociation Reagent (Gibco, ThermoFisher Scientific), washed in PBS and analyzed for CD73, CD105, CD90, CD45, CD34, CD14, CD11b, HLA-DR expression (Dominici et al., 2006) using the MSC-Phenotyping Kit (Milteny Biotec GmbH, Germany). Appropriate IgG isotype-matched antibodies and unstained cells were used as controls. Dead cells were excluded by adding 7-aminoactinomycin D (7-AAD; BD Bioscience) prior to analysis. After staining procedures, cells were acquired by FACSCanto II flow cytometer (BD Biosciences) and analyzed by FACSDiva software (BD Biosciences).

### *In Vitro* UC-MSC Multilineage Differentiation Analysis

Multipotent differentiation was assessed evaluating the ability of UC-MSCs to differentiate into adipogenic, osteogenic and chondrogenic lineages under adapted media conditions. UC-MSCs were plated at 2 × 10^4^cells/well in 24-multiwell culture plate and grown in StemPro^®^ Adipogenesis Differentiation Kit (Gibco, ThermoFisher Scientific) for 3 weeks, replacing medium every 3 days. Adipogenesis was assessed using Oil Red O (Sigma-Aldrich) staining to detect intracellular lipid vacuoles. For osteogenic differentiation UC-MSCs were plated at 10^4^ cells/well in 24-multiwell culture plate and grown in StemMACS OsteoDiff Media (Miltenyi Biotec GmbH, Germany) for 10 days, replacing medium every 3 days. Osteogenesis was assessed by alizarin red S (Sigma-Aldrich) staining to detect the deposition of intracellular calcium. Chondrogenic differentiation was assessed by plating 4 × 10^4^ cells/well in NH ChondroDiff Medium (Miltenyi Biotec GmbH, Germany) for 3 weeks, replacing medium every 3 days. Chondrogenesis was confirmed using Alcian Blue staining (Sigma-Aldrich).

### Harvest of Conditioned Medium

UC-MSCs or CSCs were cultured in MesenPRO RS™ medium or complete stem medium respectively, until cells were approximately 80% confluent, then cells were washed twice with PBS and cultured in MesenPRO RS™ medium or DMEM/F12 serum-free medium. After 48 h, conditioned media (CM) were harvested, centrifuged and filtered through a 0.22 μm syringe filter and conserved at −20°C until use.

### Cell Viability Assay

Cell viability was evaluated measuring the reduction of 3-(4,5-dimethylthiazol-2-yl)-2,5,diphenyl tetrazolium bromide (MTT; Sigma-Aldrich). The cleavage of MTT to a purple formazan product by mitochondrial dehydrogenase was spectrophotometrically quantified. In brief, treated and control cells were incubated with 0.25 mg/ml MTT solution in culture medium at 37°C for 2 h; after the removal of the medium, formazan crystals were dissolved in DMSO and absorbance measured at 570 nm (Massa et al., 2004).

### Cell Migration Assay

Cell migration was performed using BD FluoroBlock™ (BD Biosciences) inserts containing light-tight PET membrane. 2 × 10^4^ cells were plated on 8 μm inserts, previously coated with diluted (1:400) Matrigel (BD Biosciences), and allowed to migrate towards control medium (DMEM/F12) or CM. After 24 h, the inserts were stained with the fluorescent dye Vybrant carboxyfluorescein diacetate, succinimidyl ester (CFDA SE) Cell Tracer (Molecular Probes, ThermoFisher Scientific) following manufacturer’s instructions and cells migrated to the bottom of membranes were analyzed by confocal laser-scanning microscope (BioRad MRC 1024 ES) at 10× magnification, and quantified using the ImageJ software (NIH, Bethesda, MA, USA; Bajetto et al., 2006). When indicated SB225002 (Sigma-Aldrich) was used at 50 nM final concentration during both pre-treatment and experiment.

### Cytokine Array Analysis

The RayBio Human Cytokine Array 3 (Ray Biotech, Inc.) containing antibodies to detect 42 proteins was used to perform a semi-quantitative evaluation of proteins released by UC-MSCs and GBM CSCs in CM in comparison with unconditioned culture medium. One-hundred micrograms of CM or MesenPRO RS™ medium were analyzed according to manufacturer’s instructions. Signals were detected by chemiluminescence reaction with ChemiDoc Imaging system (BioRad Laboratories) and quantified using Quantity-One software (BioRad; Thellung et al., 2007).

### Co-culture of 3D Spheroids

Green fluorescent protein (GFP)-CSCs were obtained by retroviral infection with pPLAIN and stably selected by G418 antibiotic. UC-MSCs were labeled with the fluorescent dyeVybrant DiI (Molecular Probes, ThermoFisher Scientific; Thellung et al., 2011). UC-MSC spheroids were generated culturing cells in complete stem medium or seeding cells on 2% agarose coated wells in MesenPRO RS™ medium. GFP-CSCs and DiI-labeled UC-MSCs were confronted in stem complete medium, MesenPRO RS™ or both media.

### Proliferation Analysis by Dye Dilution Assay

CSCs were starved for 48 h in serum free medium and labeled with carboxyl CFDA SE (Molecular Probes, ThermoFisher Scientific) to track proliferating cells before treatments. Intra-cytoplasmic CFDA equally distributes between daughter cells, allowing discrimination of successive rounds of cell division by halving fluorescence signal. CFDA-positive cells were cultured for 72 h, harvested and washed with PBS for acquisition by FACSCanto II flow cytometer and analysis by FACSDiva software (BD Biosciences). A minimum of 50,000 CFDA-positive cells were acquired from each sample; for cell viability staining 7-AAD was added to exclude dead cells. To track proliferating cells, FACS data were analyzed using the Proliferation Wizard module of ModFit™ LT version 3.0 software (Verity Software House). Generation number within Proliferation Wizard module was set at 10. The proportions of proliferated cells at each division were obtained by ModFit analysis, which generates histograms of fluorescence intensity by applying deconvolution algorithms (Würth et al., 2013).

#### Real-Time PCR

Total RNA was extracted using the AURUM total RNA Mini Kit (BioRad), according to the manufacturer’s instructions, and reverse transcribed into cDNA using iScript cDNA Synthesis Kit (BioRad). cDNA was amplified using EvaGreen mix (BioRad) on a CF96 Touch real-time PCR (BioRad). Primers sequences were as follows:

CXCR1: forward 5′-TGCATCAGTGTGGACCGTTA-3′ and reverse: 5′-TGTCATTTCCCAGGACCTCA-3′; CXCR2: forward 5′-TGCATCAGTGTGGACCGTTA-3′ and reverse 5′-CCGCCAGTTTGCTGTATTG-3′ (Maxwell et al., 2007); GFAP: forward 5′-ATCAACTCACCGCCAACA-3′ and reverse 5′-CGACTCAATCTTCCTCTCCAG-3′; GROα (CXCL1): forward 5′-CTGGCTTAGAACAAAGGGGCT-3′ and reverse 5′-TAAAGGTAGCCCTTGTTTCCCC-3′; GROβ (CXCL2): forward 5′-ACAGTGTGTGGTCAACATTTCTC-3′ and reverse 5′-TCTGCTCTAACACAGAGGGAA-3′; GROγ (CXCL3): forward 5′- CCGAAGTCATAGCCACACTCA-3′ and reverse 5′-CTCTGGTAAGGGCAGGGACC-3′; IL-8 (CXCL8): forward 5′-CTTGGCAGCCTTCCTGATTT-3′ and reverse 5′-AACCCTCTGCACCCAGTTTT-3. Levels of target genes in each sample were normalized on the basis of GAPDH and 28S amplification and reported as relative values (Gritti et al., 2014).

#### FACS Analysis

Single CSC cultures, expressing GFP, unstained UC-MSCs, or co-cultures of both cell types were grown for 72 h, stained with allophycocyanin (APC) conjugated anti-CD182 (CXCR2; Miltenyi Biotec) in accordance with the manufacturer’s instructions. Dead cells were excluded by adding 7-AAD (BD Bioscience) prior to analysis. After staining procedures, cells were acquired by FACSCanto II flow cytometer (BD Biosciences) and analyzed by FACSDiva software (BD Biosciences), as reported (Pattarozzi et al., 2017).

### Western Blot Analysis

Cells were lysed in RIPA buffer containing the “Cømplete” protease inhibitor mixture (Roche Applied Science) for 10 min at 4°C. Nuclei were removed by centrifugation (5000 rpm at 4°C, for 10 min), and total protein content measured using Bradford assay (Bio-Rad). Proteins (20 μg) were resuspended in Laemmli buffer (2% SDS, 62.5 mM Tris, pH 6.8, 0.01% bromophenol blue, 1.43 mM 2-mercaptoethanol, and 0.1% glycerol), size-fractionated by SDS/PAGE, transferred to PVDF membrane (Bio-Rad Laboratories), and probed with primary antibodies (phospho-ERK1/2, α-tubulin, from Cell Signaling). Probed membranes were incubated with anti-IgG-horseradish peroxidase-conjugated secondary antibody; immunocomplex detection and densitometric analysis were performed using the Immobilon Western Chemiluminescent HRP Substrate and the Quantity-One Image Chemi-Doc system (all from Bio-Rad Laboratories). α-tubulin was used as internal control for protein loading (Florio et al., 2001).

### Statistical Analysis

All experiments were repeated at least three times. Data from quantitative experiments are expressed as mean ± SEM or ± SD. Statistical analysis was performed by One-way ANOVA with Dunnett’s *post hoc* test, Mann-Whitney test, two-tailed *t-test* using GraphPad Prism 5.02. Statistical significance was established at *p* < 0.05.

## Results

### Isolation and Characterization of Human Umbilical Cord Mesenchymal Stem Cells (UC-MSCs) and Glioblastoma-Derived Cancer Stem Cells (CSCs)

**Figure 1:**
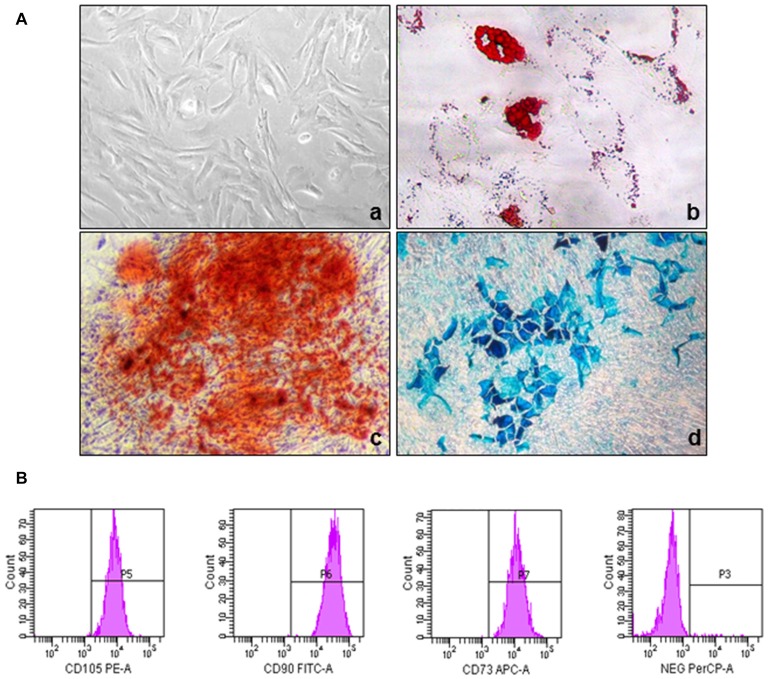
Phenotypic and functional characterization of human umbilical cord (UC)-mesenchymal stem cells (MSCs). **(A)** Representative images showing the morphology of UC-MSCs isolated from UC-1 and their differentiation into adipogenic, chondrogenic and osteogenic lineages (the same characterization was performed on all the seven established UC-MSC cultures, obtaining superimposable results): **(a)** spindle-shaped morphology of MSCs derived from Wharton’s jelly of UC; **(b)** adipocytes: red lipid vacuoles stained with the lipophilic dye Oil-Red-O; **(c)** osteocytes: alizarin red staining of calcium deposit; **(d)** chondrocytes: cartilage matrix staining with alcian blue. **(B)** Immunophenotypic characterization of UC-MSCs. Flow cytometry experiments revealed that UC-1-MSCs are positive for the typical MSC markers CD105, CD90, CD73, while lack the expression of the hematopoietic markers CD34, CD45, CD14, CD20 (all in the PerCP-A channel); histograms are representative of the results obtained with all the seven established UC-MSC cultures.

Human UC-MSCs, showing a typical spindle-shaped appearance (Figure 1, panel a), isolated from Wharton’s jelly, were selected by the ability to adhere to plastic surfaces. Cells were fully characterized according to the minimal criteria defined by the International Society for Cellular Therapy (Dominici et al., 2006). Flow cytometry analysis of cell surface antigens indicated that these cells express CD73, CD90 and CD105, which are considered typical MSC markers, while they were negative for CD11b, CD14, CD34, CD45 and HLA-DR (Figure 1B). UC-MSCs showed multilineage differentiation ability acquiring adipogenic, osteogenic and chondrogenic phenotypes. These features were demonstrated by accumulation of Oil Red O-stained lipid droplets (adipocytes, Figure 1A, panel b), enhanced mineralization, evidenced by Alizarin Red staining (osteocytes, Figure 1, panel c) and Alcian blue staining in 2D test (chondrocytes, Figure 1A, panel d). Superimposable results were obtained analyzing cells from all the seven UC cultures studied. No neutral lipid droplet staining, mineralization or chondrogenic differentiation occurred when cells were cultured in control medium (data not shown), and human fibroblast never generated adipocyte- or osteocyte-like cells under the same differentiating culturing conditions (data not shown).

**Figure 2:**
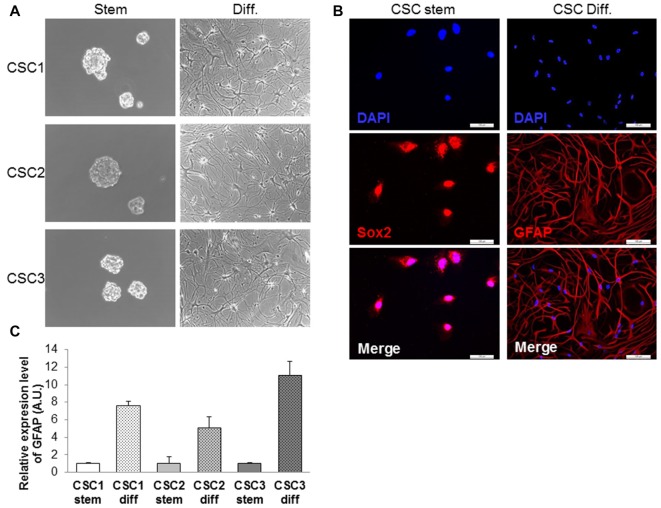
Characterization of glioblastoma (GBM) cancer stem cells (CSCs). **(A)** Morphological appearance of CSCs grown as floating spheres in complete stem medium (Stem) and as monolayers after induction of differentiation in serum-containing medium (Diff.). **(B)** Representative immunofluorescence images of Sox2 (red nuclear staining) and GFAP (red cytoskeleton staining) expression in CSCs before and after induction of differentiation in serum-containing medium. Nuclei were counterstained with DAPI (blue). Scale, 100 μm. **(C)** Quantitative RT-PCR of GFAP mRNA levels in CSC1, CSC2 and CSC3 cells cultured in complete stem medium or after differentiation. Normalized values are referred to stem condition. Values are expressed as the mean of three experiments ± SD.

CSCs were isolated from three different human GBMs (named CSC1-2-3) by their ability to grow as neurospheres in stem cell permissive medium, containing bFGF and EGF in the absence of FBS (complete stem medium, see “Materials and Methods” section; Figure 2A). Growing the cells in the absence of growth factors, a marked reduction in the ability of CSCs to proliferate *in vitro* was observed that was completely abolished culturing the cells in plain DMEM/F12 medium (see Supplementary Figure S1). In the absence of growth factors, CSCs also lose spherogenic ability (Supplementary Figure S2). However, when growth factors were replaced with FBS, GBM CSCs are induced to differentiate, evidenced by morphological change, surface adhesion, and growth as monolayer (Figure 2A). CSCs express stemness-associated markers, such as SOX2 (Figure 2B), nestin and CD133 (data not shown), while differentiated cells show increased expression of the astrocytic protein GFAP, as shown in immunofluorescence experiments (Figure 2B). These results were confirmed by qRT-PCR, showing increased GFAP mRNA levels in differentiated cells from all the three GBMs as compared to the respective CSCs (Figure 2C). All GBM CSC cultures used in this study have been evaluated in previous experiments for the ability to form tumors when orthotopically xenotransplanted in the brain of NOD/SCID mice, confirming the retaining of tumorigenic activity even after prolonged *in vitro* growth (Corsaro et al., 2016; Falcone et al., 2016).

### Reciprocal Tropism between UC-MSCs and CSCs and *in Vitro* Invasion

Several studies reported that tumor-derived soluble factors mediate the chemotactic tropism of MSCs toward GBM cells (Bexell et al., 2010). We used a spheroid confrontation invasion assay as model to monitor migration ability of the different cell types, since cells grown as multicellular spheroids closely recapitulate the *in vivo* biological behavior of solid cancers. CSC-enriched cultures from all the three GBM analyzed (CSC1, CSC2, CSC3) were infected with a retrovirus expressing the GFP protein to trace cells through the experiments, while UC-MSCs were marked out with the red fluorescent lipophilic dye DiI (Thellung et al., 2011). CSCs, cultured in stem cell-permissive medium, grow as spheroids (Griffero et al., 2009; Gatti et al., 2013); in contrast, UC-MSCs grow as adherent cells in standard culture conditions. To generate multicellular spheroids of UC-MSCs, these cells were grown in mesenchymal defined medium on agar feeder, to prevent plastic adhesion or, alternatively, in complete stem cell medium, in which both CSCs and UC-MSCs grow as spheroids (see “Materials and Methods” section). Co-culture experiments using spheroids of both cell types were performed in the different culture conditions (mesenchymal defined medium on agar-coated plates or without coating) and their migration and interactions were documented by confocal microscopy.

**Figure 3:**
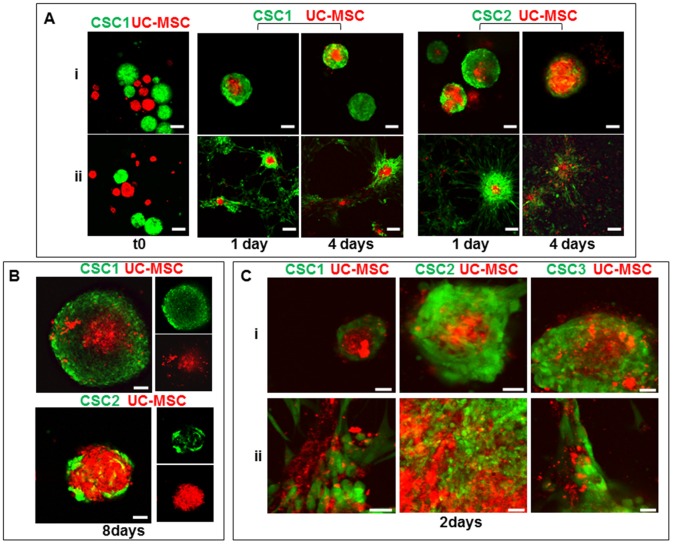
Co-culture of GBM CSCs and UC-MSCs in 3D spheroids (images of UC-2-MSC cultures are reported, but the same results were obtained also using UC-3-MSC). **(A)** Time-dependent integration of 3D spheroids of green fluorescent protein (GFP)-CSC1, GFP-CSC2 (green) and DiI-stained UC-MSCs (red), co-cultured in mesenchymal defined medium in the presence **(i)** or absence **(ii)** of agar coating, analyzed by confocal microscopy after 1 and 4 days. Scale bar, 160 μm. **(B)** Analysis by confocal microscopy after 8 days of co-culture of spheroids of GFP-CSC1 or GFP-CSC2 (green) and spheroids DiI-stained UC-MSCs (red) formed in complete stem cell medium. Scale bar, 50 μm. In small images only green or red channel of mixed spheroid are shown. **(C)** Analysis by confocal microscopy after 2 days of co-culture of GFP-CSC1, GFP-CSC2, GFP-CSC3 (green) and DiI-stained UC-MSCs (red) cells co-cultured in complete stem cell medium **(i)** or mixed complete stem medium and defined mesenchymal medium 1/1 **(ii)**. Scale bar, 50 μm.

When, confronted in the absence of cell adhesion to the plastic support (agar coating), single spheroid of CSCs and UC-MSCs entered in close contact after only 1 day of co-culture, and a red core (UC-MSCs) was appreciable within green aggregates of both CSC1 and CSC2 (Figure 3, panel Ai). After 4 days, the majority of UC-MSCs and CSC1 (or CSC2) appeared to be strongly intertwined with each other in the same spheroid structure (Figure 3, panel Ai). In the absence of agar, spheroids attached to the plastic surface and both CSC1 and CSC2 cultures strictly adhered to UC-MSCs, as observed in the three-dimensional spheroids (Figure 3, panel Aii). The confrontation of UC-MSC and CSC spheroids in complete stem medium showed a similar biological behavior. Acquisition of laser confocal images after 1, 2 (not shown) and 8 days (Figure 3B) showed a bidirectional tropism. In particular, DiI-labeled UC-MSCs migrated into GFP-expressing CSC spheroids, as well as invasion of the red UC-MSCs spheroid by green CSCs was evident, indicating an active reciprocal tropism of the two cell types in both culture media (Figure 3B). Similar results were obtained when dispersed single cells from UC-MSCs and GFP-CSC1 or GFP-CSC2 or GFP-CSC3 were seeded together either in complete stem medium or in the mixture of complete stem cell medium plus defined mesenchymal medium (1:1). After 2 days of co-culture, both cell types interacted forming mixed red-green spheroids in complete stem medium (Figure 3, panel Ci) or partially adherent spheroids in mixed medium (Figure 3, panel Cii).

These results suggest that UC-MSCs and GBM-CSCs are able to migrate toward each other forming mixed spheroids independently from the culture conditions used, and that their integration does not produce apparent phenomenon of cell death, even after 8 days of co-culture.

### Reciprocal Control of Proliferation between GBM CSCs and UC-MSCs

**Figure 4:**
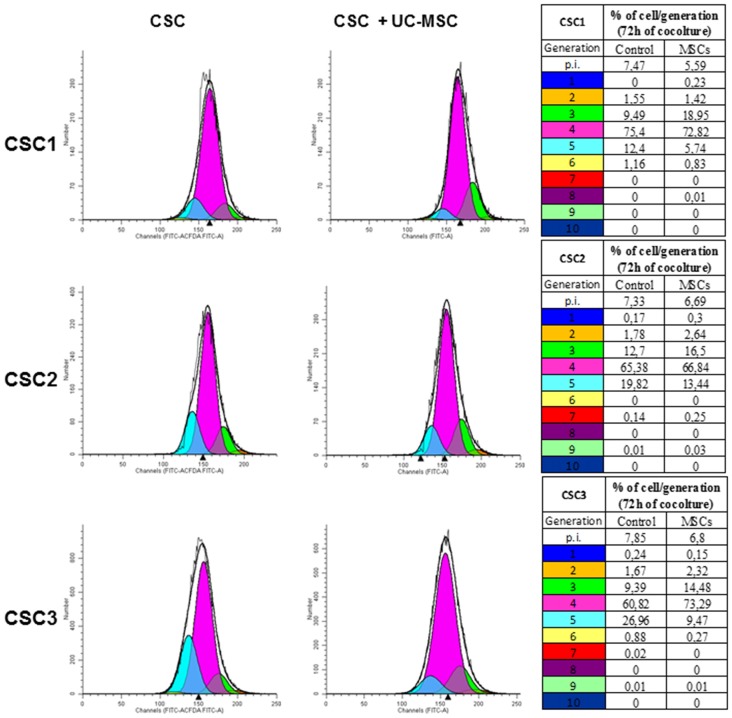
UC-MSC co-culture reduces GBM CSC proliferation rate. The proliferation rate of CSC1, CSC2 and CSC3 was tracked by carboxyfluorescein diacetate, succinimidyl ester (CFDA SE) dye dilution and analyzed by flow cytometry. The distribution of CSC1, CSC2 and CSC3 cells among generations after 72 h of co-culture with UC-MSCs (1:1) is depicted in histograms, each curve represents a new generation that occurred, starting from the parental cells (blue curve). The quantification of the data is summarized in the corresponding tables. Proliferation index (p.i.) is reported on the top of tables representing the sum of cells in all generations divided by the number of original parent cells. Representative data of three independent experiments using different UC-MSCs (1, 3 and 4) are shown.

To determine whether direct contact between GBM CSCs and UC-MSCs is required to interfere with tumor growth, CSC proliferation was evaluated using CFDA-SE dye dilution assay analyzed by flow cytometry, that allows cell division tracking as sequential halving of initial fluorescence in daughter cells. CSC1, CSC2 and CSC3 were labeled with CFDA-SE and then cultured in the absence or presence of UC-MSCs, in stem complete medium for up to 72 h. Proliferation analysis was performed by FACS every 24 h, evaluating the decline of CFDA-SE fluorescence due to dye distribution in daughter cells (Figure 4). CSC growth rate after 72 h was significantly reduced when co-cultured with UC-MSCs, as evidenced by the delay in fluorescence leftward shift, as well as by reduction of the proliferation index (p.i.), as compared to control CSC cultures. In detail, in the presence of UC-MSCs, the slow-down of CSC1 and CSC3 proliferation occurred as a reduction of the percentage of cells that engaged the third and the fifth cell division, respectively, and was quantified by the decrease of the p.i. from 7.5 to 5.6 for CSC1 and from 7.8 to 6.8 for CSC3 (Figure 4). In CSC2 the presence of UC-MSCs had a minor effect, with a reduction of p.i. from 7.3 to 6.7. Moreover, CSC proliferation kinetics was modified by co-culture with UC-MSCs: although, as observed in control cells, proliferating CSCs progress up to the fifth generation after 72 h, this occurred in a significant lower percentage of cells (5.74 vs. 12.4, 13.4 vs. 19.82 and 9.47 vs. 26.96 for CSC1, CSC2 and CSC3, respectively). Notably, a similar pattern of cell division slowdown was observed in all the cultures, although slightly less pronounced in CSC2 (Tables in Figure 4). Taken together these data suggest that the direct interaction with UC-MSCs induces inhibition of CSC proliferation.

**Figure 5:**
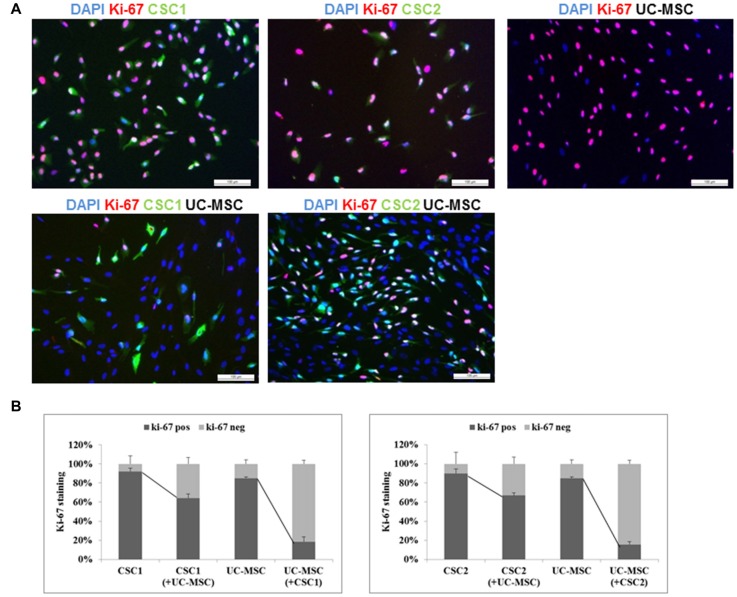
Modulation of the growth fraction of GBM CSCs expressing GFP and UC-MSCs as single culture or co-culture systems (images of UC-5-MSC cultures are reported, but the same results were obtained also using UC-6-MSC). **(A)** Representative immunofluorescence staining for Ki-67 (red) in GFP-CSC1 (green), GFP-CSC2 (green), UC-MSCs (not stained) or in GFP-CSC1/UC-MSC and GFP-CSC2/UC-MSC co-cultures after 2 days. Nuclei are counterstained with DAPI (blue). Scale bar, 100 μm. **(B)** Quantification of Ki-67 staining was obtained using ImageJ Software. Total number of cells was identified by DAPI nuclear staining. The number of UC-MSCs was obtained by difference from the number of blue stained cells and those green stained (CSCs). CSC Ki-67-positive are identified by triple staining green/red/blue; CSC Ki-67-negative by double staining green/blue; UC-MSC-Ki-67-expressing cells show double staining red/blue and UC-MSC-Ki-67 negative only blue staining. Histograms represent the % of Ki-67-expressing cells ± SEM among the total number of cells counted in four different fields for each conditions, identified by DAPI nuclear staining.

To better understand this phenomenon, we evaluated by immunocytofluorescence the expression of Ki-67 nuclear antigen, an index of cell proliferation, in GFP-CSC1, GFP-CSC2 and UC-MSCs monocultures or after their co-culture for 48 h in complete stem cell medium (Figure 5A). Histograms reported in Figure 5B show that, after 2 days of cultures, Ki-67 positive stain (red) was present in 92 and 90% of GFP-CSC1 and GFP-CSC2, respectively (green); in UC-MSC (evidenced by blue nuclei without cytosol green stain), Ki-67 antigen was detected in 85% of the cells. However, when co-cultures were established, a significant reduction of Ki-67 antigen expression occurred in GBM CSCs (from 92% to 63% in CSC1 and from 90% to 67% in CSC2; Figure 5B), demonstrating that in the presence of UC-MSCs both CSC cultures reduced the proliferation rate, in line with the FACS results described in Figure 4. A remarkable reduction of Ki-67 was also observed in UC-MSCs: in the presence of CSC1 or CSC2, Ki-67 expression was detected in only 17% or 15% of UC-MSCs, respectively (Figure 5B). The morphology of nuclei counterstained with DAPI confirmed the absence of apoptosis in both cell types.

These results clearly show that direct cell-to-cell interaction of co-cultured CSCs and UC-MSCs causes a reduction of proliferation rate of both cell types.

### UC-MSC Conditioned Media Induce CSC Growth through ERK1/2 and Akt Activation

**Figure 6:**
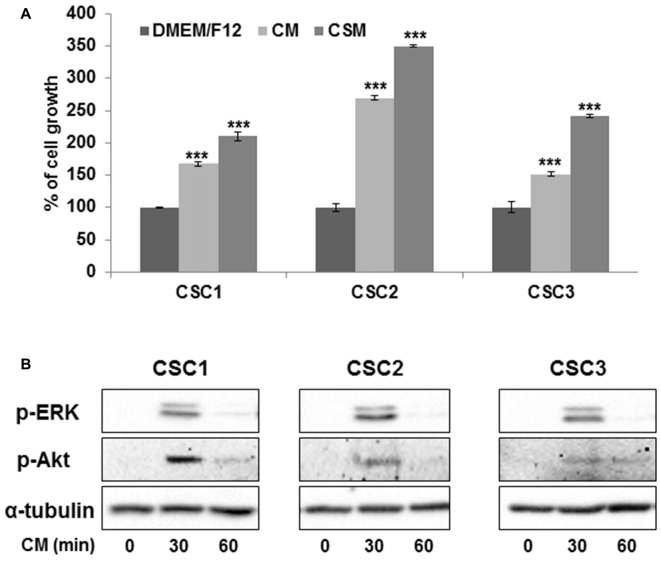
UC-MSC conditioned medium (CM) promotes the growth of GBM CSCs through ERK1/2 and Akt activation. **(A)** CSC growth was analyzed after 72 h of culture in the absence (CTR) or presence of UC-MSC-derived CM by MTT assay. Columns represent the mean of seven different CM tested ± SD (the effects of individual CM are reported in Supplementary Figure S3). Statistical analysis was performed with One-way ANOVA *p* < 0.0001 with *post hoc* analysis by the Dunnett’s test ***p < 0.001. **(B)** Representative Western Blot analysis showing the expression of phospho-ERK1/2 and phospho-Akt in control (time 0) CSCs and after stimulation with UC-MSCs CM for 30 min or 60 min. Hybridization with total α-tubulin was used as loading control.

To identify possible UC-MSC-dependent factors that may influence CSC proliferation, we evaluated the growth of CSC1, CSC2 and CSC3 in the presence of UC-MSC-derived CM collected from seven different UC-MSC cultures after 48 h of growth in serum free DMEM/F12 medium. Proliferation was assessed after 72 h from the addition of CM, using the MTT assay. Unexpectedly, the results evidenced that all UC-MSC CM induced a consistent increase in cell proliferation, averaging +69% in CSC1, +167% in CSC2, and +51% in CSC3 in comparison to control cells grown in DMEM/F12 medium (Figure 6A, see Supplementary Figure S3 for the effects of individual media). As further control, we tested the effects of UC-MSC-derived CM on the growth of UC-MSCs without observing any modification of proliferation rate (data not shown), while CM from the three CSC cultures did not induce UC-MSC proliferation (Supplementary Figure S4). Thus, a monodirectional regulation of cell proliferation was only observed, induced by soluble factors released by UC-MSCs and acting on GBM CSCs.

To investigate the intracellular signaling mediating the CSC proliferation induced by UC-MSC CM, we quantified by Western blotting the phosphorylation/activation status of ERK1/2 and Akt kinases, which are critical mediators of pathways regulating CSC growth and survival (Griffero et al., 2009; Würth et al., 2013). A strong transient phosphorylation of ERK1/2 was detected in all CSC cultures after 30 min of exposure to UC-MSC CM, which returned to basal levels after 60 min (Figure 6B). Likewise, the phosphorylation of Akt in response to UC-MSC CM was maximal after 30 min, and declined, although still slightly detectable, after 60 min in CSC3 culture (Figure 6B). Alpha-tubulin levels, used as internal control, remained unchanged in all the experimental conditions. These results suggest that UC-MSCs release soluble factors which exert a stimulatory activity on CSC proliferation through a transient activation of both ERK1/2 and Akt pathways.

### Cytokine Profile in UC-MSC and in CSC Conditioned Media

**Figure 7:**
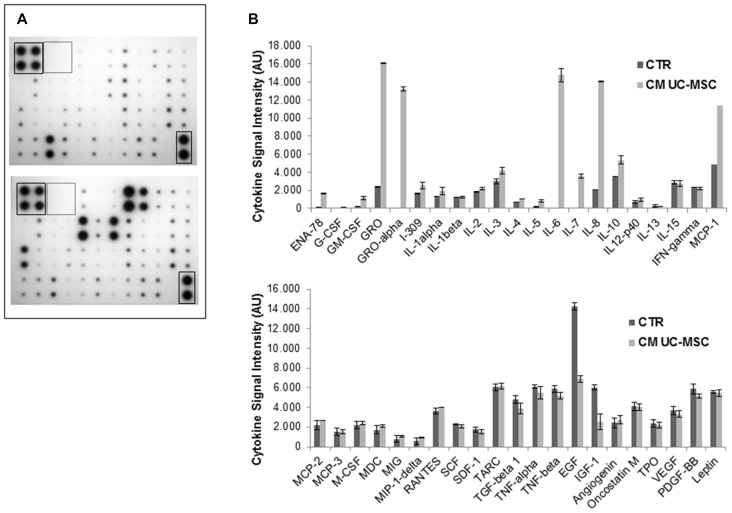
Profile of cytokines secreted into the CM of UC-MSCs. The expression level of a panel of cytokines was analyzed using a human cytokine array in the CM from three independent UC-MSCs (3, 4 and 5) harvested after 48 h of culture. The different CM analyzed induced highly reproducible results. **(A)** Representative blots of cytokines found in MesenPRO medium (upper panel) and in mesenchymal medium (lower panel); boxes represent the positive (solid line) and negative (dotted line) controls, respectively. **(B)** Densitometric analysis for quantification of cytokine spots, after subtraction of cytokine spots found in mesenchymal medium. Results are reported as arbitrary units (AU) of signal intensity in controls (CTR) and CM of UC-MSCs. Data are normalized after background subtraction.

Chemokines, and cytokines in general, are major players mediating MSC migration toward tumor tissues. The contrasting results we evidenced by the co-culture of UC-MSCs and CSCs and the activity of soluble components of UC-MSC CM, prompted us to analyze UC-MSC and CSC cytokine secretome using a commercial cytokine antibody array (Figure 7A). To evaluate the cytokine profile released from UC-MSCs we collected the supernatants after 48 h of culture in standard conditions (MesenPRO medium) and used this uncultured medium as control. The results evidenced the presence of multiple chemokines and cytokines, some of which were already components of the MesenPro medium and others specifically produced by UC-MSCs (Figure 7A). To quantify the amount of cytokines released by UC-MSCs, spots on membranes were analyzed by densitometry and plotted after subtraction of the respective spots detected in MesenPRO medium (Figure 7B). The cytokines released at higher levels by UC-MSCs were GRO, GROα, IL-6, IL-8 and MCP-1 (Figure 7B). UC-MSC CM, collected in serum free medium DMEM/F12 (in which no cytokines are present), showed a similar cytokine content profile indicating that this activity is independent from the culture conditions (data not shown). Importantly, the cytokines secreted in higher amounts by UC-MSCs are mainly characterized by a positive activity on angiogenesis, inflammation, cell migration and tumorigenesis, rather than playing antiproliferative or cytostatic roles.

**Figure 8:**
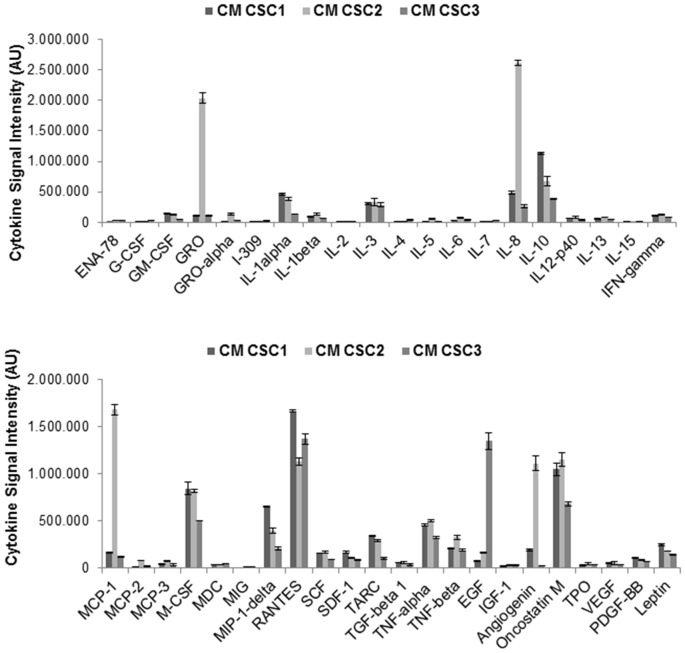
Cytokines profile in CSC1, CSC2 and CSC3 cells. CM were collected from CSC1, CSC2 and CSC3 cultures after 48 h in serum free medium. Cytokine antibody arrays were performed and quantification analysis was done by densitometry of cytokine spots, reported as AU of signal intensity. Data are normalized after background subtraction.

As far as CSCs, the pattern of cytokines secreted was analyzed using the same antibody array. CSC CM was collected after growing CSC1, CSC2 and CSC3 in serum free medium for 48 h. Results showed that CSCs secrete a different pattern of cytokines from UC-MSCs, being non-always overlapping between CSC1, CSC2 and CSC3 (Figure 8). The simultaneous screening of different cytokines demonstrated that the most represented molecules found in the CM from all CSC cultures include oncostatin M, angiogenin, TNF-α and β, TGF-β1, RANTES, MCP-1, MIP-1δ, M-CSF, IL-10, IL-8, IL-1 α and β, IL-3 and GRO (Figure 8). These experiments also highlighted that CSC2 secrete higher level of GRO, IL-8, MCP-1 and angiogenin as compared to CSC1 and CSC3, which, in contrast, showed a more homogeneous cytokine secretome. Notably, the secretion of these cytokines (GRO, IL-8 and MCP-1) was observed at high levels in both CSCs and UC-MSCs.

**Figure 9:**
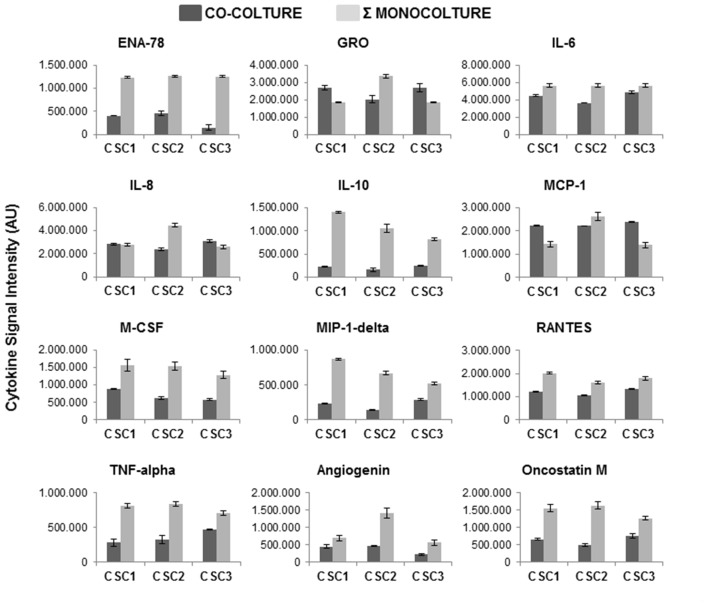
Effects of co-culture of CSC1, CSC2 and CSC3 with UC-MSC-CM on cytokine release. CM were collected from CSC1, CSC2 and CSC3 cultures after 48 h of co-culture with UC-6-MSC in DMEM/F12 medium. Data are compared with the densitometry results obtained by the sum of the cytokines released by CSCs and UC-MSCs mono-cultures. The same protein arrays described in Figures 7, 8 were used, but for sake of clarity, only the values of cytokines that resulted, modified in the co-cultures are reported. Data are the average of two determinations.

As shown in Figure 9, the pattern of cytokine released by both CSCs and UC-MSCs was changed when the two populations were co-cultured. The analysis was performed by comparing the levels of cytokines released in the medium for 48 h in co-culture with the levels obtained by the sum of cytokines released by CSCs and UC-MSCs grown separately. Interestingly, while co-culture did not modify the release of some peptides (i.e., SCF, SDF-1, GRO-α, IL-7) independently from which cell type mainly released the cytokine, a general inhibitory pattern was observed for most of the secreted proteins. In particular, an inhibitory trend was observed in the co-culture as far as ENA-78, IL-6 and IL-10 mainly produced by UC-MSCs, and M-CSF, MIP-1δ, RANTES, TNF-α, angiogenin, and oncostatin M, mainly released by CSCs. A more complex pattern was observed for GRO, MCP-1 and IL-8, that are released at high levels by UC-MSCs and CSC2. In the coculture UC-MSC/CSC2 the release of all these peptides was inhibited, while it was potentiated co-culturing UC-MSC and CSC1 or CSC3, as far as GRO, MCP-1, but remained unchanged for IL-8. Validation of chemokine expression in UC-MSC and CSC1-3 was performed for few relevant genes (in particular, the GRO isoforms and IL-8) by qRT-PCR, confirming the expression of all genes (Supplementary Figure S5).

### CXCR2 Inhibitor Mediates Anti-Proliferative and Anti-Migratory Effects in CSC2 and CSC3 Co-cultures with UC-MSC CM without Affecting Their Spheroid Formation and Invasion Capacity

**Figure 10:**
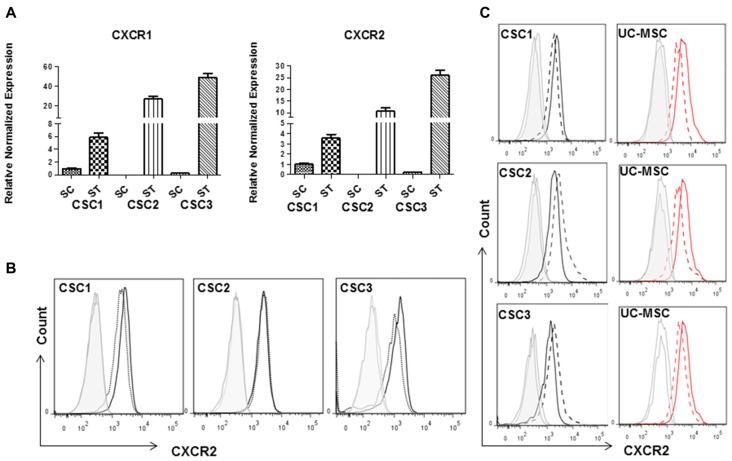
Expression levels of CXC chemokine receptor 1 (CXCR1) or CXCR2 in CSCs in basal or starved culture conditions. **(A)** Total RNA was isolated from CSC1, CSC2 and CSC3 cultured in stem cell medium (SC) or starved (ST) for 48 h, and quantitative RT-PCR for CXCR1 or CXCR2 was carried out. Expression values of ST were normalized to CSC1 SC levels (taken as 1), and data are expressed as the mean of three experiments ± SD. Data derived form the average of two evaluations using independent mRNA extraction and cDNA amplification. **(B)** FACS analysis reporting CXCR2 expression in CSC1–3 cultures, grown in stem medium (black lane) or in serum free medium for 48 h (dashed black line); Ab isotype signal is depicted by gray lines. At protein levels the culture conditions did not affect CXCR2 expression. **(C)** Histograms showing CXCR2 expression in CSCs (black line) or UC-MSCs (dashed red line) cultured individually or co-cultured (dashed lines) for 3 days. Ab isotype are shown in both conditions (gray lines). The expression of CXCR2 in both CSCs and UC-MSCs was not modified by co-culturing.

IL-8, GROs and GROα which represent the chemokines mainly involved in cell migration and angiogenesis, share CXCR2 as common receptor. We used the specific CXCR2 antagonist SB225002 to study the role of the activation of this receptor in the proliferative activity exerted by UC-MSC CM on CSCs, and in the CSC/UC-MSC spheroid formation and invasion. First, we demonstrated that both CXCR1 and CXCR2 mRNAs are expressed in all CSCs (Figure 10A). Their expression was clearly evident in cultures maintained under growth factor starved conditions (ST; Figure 10A), while a weaker expression was detected in CSC1 and CSC3 maintained in standard conditions (stem cell medium, SC, Figure 10A) and it was almost absent in CSC2 culture. These results were confirmed at protein level, by FACS analysis (Figure 10B) in which CXCR2 protein expression was detected in all CSCs and barely modified by changing the culture conditions.

**Figure 11:**
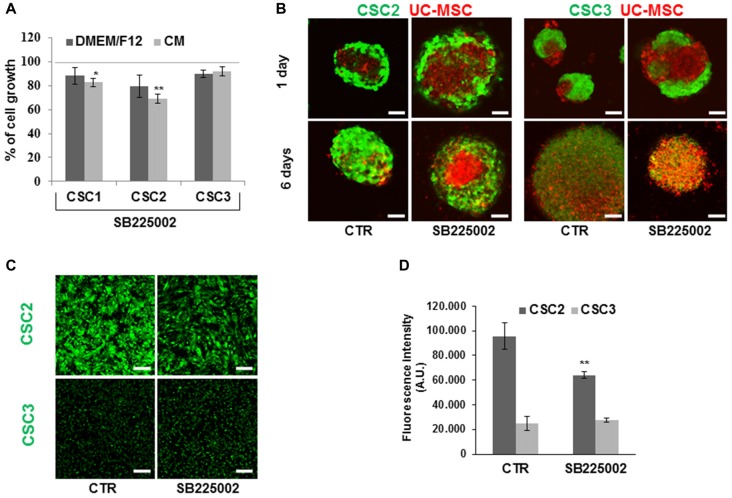
Effects of the CXCR2 antagonist SB225002 on CSC growth dependent or independent from CM, spheroid CSC/UC-MSC co-culture formation and cell migration. **(A)** CSC1, CSC2 and CSC3 were pretreated for 48 h in standard growth condition with SB225002 (50 nM) for 48 h, and then were challenged with seven different UC-MSC CM for 72 h or in serum free medium (DMEM/F12), in the presence or in absence of SB225002, before MTT assays. Histograms represent the average of the effects of all the seven distinct CM used in this study ± SEM. Data are expressed as % of cell growth in CM or DMEM/F12 in absence of the inhibitor taken as 100%. Statistical analysis was performed using the Mann-Whitney test. *P < 0.05 and **P < 0.01. **(B)** Co-culture of GFP-CSC2 and GFP-CSC3 (green) and DiI-stained UC-5-MSC (red) spheroids for 1 and 6 days in the presence or in absence of SB225002 (similar results were also obtained using UC-6-MSC). Images were acquired by confocal laser microscopy. Scale bar, 53 μm. **(C)** Representative images of transwell migration assays on GFP-CSC2 and GFP-CSC3 untreated (CTR) or treated with SB225002 (50 nM). Scale bar, 160 μm. **(D)** Cell migration through transwell inserts was quantified measuring the intensity of fluorescence of migrated cells from four different confocal fluorescence images. Histograms represent the mean fluorescence intensity expressed as AU ± SD. Statistical analysis was performed by the unpaired *t*-test **P < 0.01.

CSC1, CSC2 and CSC3, pretreated with SB225002 for 48 h in complete stem medium, were challenged for 72 h with UC-MSC CM individually collected from seven independent cultures, in the presence or absence of the CXCR2 inhibitor, and cell growth was analyzed by MTT assay. CXCR2 blockade significantly reduced cell proliferation in CSC2 and, although at lower extent, CSC1 and CSC3 cultures (Figure 11A). These differential data likely reflect individual features of CSCs derived from different GBMs, with CSC2 growth being more dependent on CXCR2 signaling than the other cells. In fact, the possible “addiction” of CSC2 for CXCR2 ligands was also suggested by the higher basal production of IL-8 and GRO as compared to CSC1 and CSC3.

To delve deeper into the mechanisms of the interaction and invasion of spheroids formed by the different cell types, we analyzed by confocal laser microscopy GFP-CSC2 and GFP-CSC3 cells co-cultured with UC-MSCs, pretreated with SB225002 for 48 h, after 1 and 6 days in complete stem cell medium. The presence of SB225002 did not modify neither spheroid formation nor invasion of CSC2 and CSC3 or UC-MSCs within spheroids (Figure 11B), even after 6 days of co-culture. Nevertheless, the inhibition of CXCR2 activity markedly reduced CSC2 migration (33% of reduction), as assayed by transwell migration assays, whereas, in the same experimental conditions, it did not affect CSC3 migration (Figures 11C,D). Notably, CXCR2 expression in co-culture conditions was only marginally modified in both all GBM CSCs and UC-MSC (Figure 10C), suggesting that the lack of effects of the CXCR2 inhibitor on spheroid invasion and cell migration is not dependent on receptor dynamics.

These results suggest that CXCR2 activation by secreted ligands controls CSC growth and migration, although individual differences are detectable among cultures derived from different GBMs. Conversely, SB225002-mediated inhibition of CXCR2 did not influence spheroid formation and invasion ability of both CSC2 and CSC3 co-cultures with UC-MSCs.

## Discussion

In the past years, MSCs demonstrated glioma-targeting behavior after transplant into rat brain. MSCs were reported to migrate along the corpus callosum toward the area where GBM was established in the contralateral hemisphere or in distant microsatellites (Nakamura et al., 2004), and to home into glioma after injections into the ipsilateral and contralateral carotid arteries (Nakamizo et al., 2005). Moreover, *in vitro* studies demonstrated MSCs to possess direct anti-tumor properties, impairing the growth of GBM cell lines and patient-derived primary GBM cultures, while the co-injection of MSCs and GBM cells resulted in a significant reduction of volume and vascularization of the tumor developed *in vivo* (Ho et al., 2013).

However, contrasting observations were also reported, supporting the possibility that MSCs actually promote tumor development and growth through stimulation of cancer cell proliferation or favoring angiogenesis and immunosuppressive activity (Lazennec and Jorgensen, 2008; Klopp et al., 2011). Most of the studies concerning the trophic or pro-apoptotic properties of MSCs toward tumors were carried out in GBM established cell lines, and only few reports focused on MSC interaction with human CSCs, even though this rare tumor cell subpopulation, responsible of tumor recurrence and drug resistance, represents the main pharmacological target to eradicate neoplasms (Shinojima et al., 2013; Liu et al., 2014).

In the present study, we investigated how specific cellular interactions between CSCs and UC-MSCs affect *in vitro* proliferation of both populations. We identified different and sometimes contrasting effects deriving from the interaction between UC-MSCs and three GBM-derived CSC cultures as far as their ability to control cell proliferation, invasion and migration.

Using 3D spheroid invasion assay, we show that UC-MSCs and CSCs possess a reciprocal tropism, with both cell types able to infiltrate the counterpart spheroid, and to form compact spheroids including closely integrated cells of both populations. This activity was observed in different experimental conditions and was independent from the culture medium used in the assay, considering that the two cell types normally grow in the presence of different medium formulations. This process was particularly evident in CSC2 culture. This observation is of utmost relevance considering that the lethal clinical outcome of GBM mainly resides in the invasiveness of glioma cells and that mesenchymal cells are component of the tumor microenvironment and may modulate this migratory capacity (Behnan et al., 2014).

In addition, our data suggest that prolonged co-culture of UC-MSCs and CSCs did not induce apparent apoptosis or necrosis, in both cell populations within spheroids. However, the direct co-culture caused a significant inhibition of both UC-MSC and CSC proliferation rate. These results indicate that not only UC-MSCs influence GBM CSC proliferation but also CSCs might influence the growth of UC-MSCs, possibly to gain support for their invasiveness into surrounding tissue.

Conversely, the anti-proliferative effect of UC-MSCs was not observed when CSC proliferation was evaluated after exposure to UC-MSC CM, which rather increased CSC proliferation, associated with a transient activation of ERK1/2 and Akt intracellular signaling. The analysis of cytokine content within the CM of UC-MSCs demonstrated a strong enrichment of important tropic and trophic factors, including IL-6 and the chemokines ENA-78 (a.k.a CXCL5), IL-8 (CXCL8) and the GRO-related peptides (CXCL1, CXCL2 and CXCL3). Importantly, CSCs, and in particular the CSC2 culture, also release IL-8 and GROs. All CSCs express mRNA for CXCR1 and CXCR2 when cultured in starved conditions (w/o cytokines and growth factors), while in standard culture conditions (stem cell medium) only weak mRNA expression was detected in CSC1 and CSC3 but not in CSC2. This evidence supports the activation of an autocrine/paracrine loop in GBM CSCs, in which the abundant production of IL-8 and GRO over-activates and then down-regulates CXCR2 mRNA expression in standard culture conditions (paradoxically the lower levels of expression may reflect the higher activation of the pathway) whereas the receptor expression level increases only after the removal of chemokines from the medium (cell starvation). A similar regulatory mechanism has been described for another chemokine receptor (CXCR4) in GBM CSCs (Gatti et al., 2013). This mechanism was confirmed analyzing CXCR2 expression at protein level by FACS that indeed confirmed the significant presence of CXCR2 in both CSCs and UC-MSCs. Only marginal changes were observed in the expression of CXCR2 in CSCs and UC-MSCs when co-cultured.

CSC proliferation induced by UC-MSC CM was mediated, at least in part, by the activation of CXCR2. In fact, in the presence of the CXCR2 antagonist SB225002, UC-MSC CM-dependent cell proliferation was partially inhibited in all CSCs, but this effect was more evident in CSC2 cells which spontaneously release high levels of CXCR2 ligands, IL-8 and GRO. The higher sensitivity of CSC2 to CXCR2 inhibition was also demonstrated by SB225002 ability to reduce CSC2 migration, while only minor effects were observed in CSC3, in agreement with the lower production of CXCR2 ligands. These results, while confirming the expected individual differences within GBM CSC cultures, which reflect the distinct biological behavior and molecular heterogeneity (mutations in EGFR, IDH1, IDH2, etc.) of the original tumor, highlight how some GBM CSCs can be dependent on chemokines for proliferation and migration, and that these could be supplied not only by autocrine mechanisms but also by cells within the tumor stroma (or the CSC niche), including MSCs. However, we have to acknowledge that our experimental model has some limitations since, although it analyzes the interactions between CSCs and MSC in a 3D environment, it does not include specific CNS cell populations such as astrocytes and microglia. Importantly, these cells are activated during GBM development resulting in high production of cyto/chemokines. Thus, although the *in vivo* situation is surely more complex than that reproduced in our model, the establishment of the role of the cytokine milieu in the control of GBM progression, as we report, is of extreme relevance. Further studies, more specifically addressing the involvement also of astrocytes and microglia, will complete our observations.

Similar effects of cytokines were observed in different tumor models. For example in ovarian cancer, ovarian MSCs release IL-6 to promote proliferation and colony formation (Ding et al., 2016) with a mechanism similar to that we observed with CXCR2 ligands. Nevertheless, in our study SB225002 did not interfere with CSC or UC-MSC spheroid formation and invasion in the co-culture experiments. While we cannot exclude that the close adhesion occurring in spheroids among UC-MSCs and CSCs prevents the access of the inhibitor inside the spheroid, we hypothesize that the physical contact between GBM CSCs and UC-MSCs, likely regulated by adhesion molecules, modifies the release of or response to chemotactic factors.

Thus, a completely different *scenario* can be drawn as far as UC-MSC modulation of GBM CSCs proliferation, according to the occurrence of a direct interaction between the different cell populations (mainly showing antiproliferative activity, independent from cyto/chemokine release and CXCR2 activation), or when the interaction is mediated by soluble factors, and CXCR2 ligands in particular, which determine an opposite response (CSC proliferation and activation of migration).

GROs and IL-8, highly secreted by both UC-MSCs and CSCs, share the same receptors CXCR1 and CXCR2 with other chemokines (ENA-78, GCP-2 and NAP-2). CXCR2 exerts its canonical activity regulating neutrophil migration from bone marrow and their recruitment into sites of inflammation. In addition, CXCR2 has been identified as a fundamental mediator in tumorigenesis not only in malignant tumors (Liu et al., 2016) but also in pituitary adenomas (Barbieri et al., 2011). High expression of IL-8 and its receptors in tumor microenvironment might support tumor progression via the establishment of pro-inflammatory signaling in tumor cells, promoting proliferation, angiogenesis, migration and invasion of cancer cells and, through paracrine signals, acting also on stromal and endothelial cells (Campbell et al., 2013).

GROs were initially isolated from the CM of a melanoma cell line (Richmond and Thomas, 1988) and their role in promoting tumorigenesis was identified in several tumors. Downregulation of CXCR1 and CXCR2, by interfering siRNA, inhibits melanoma tumor growth and cell invasion (Singh et al., 2010); similarly RNAi of GROα suppresses tumor growth in hepatocellular carcinoma (Han et al., 2016). In GBM cell lines, GROα promotes tumor growth, *in vitro* cell motility and invasiveness and enhances tumor cell spread *in vivo* (Zhou et al., 2005). This chemokine was also reported to activate the recruitment of bone marrow MSCs into diffuse-type gastric cancer stroma and the inhibition of migration in tumor microenvironment induced by the CXCR2 antagonist SB225002, decreases tumor volume and metastasis (Kasashima et al., 2016). GROα and β also facilitate the development of lung metastases and chemoresistance in breast cancer through paracrine activation of CD11b^+^/Gr1^+^ myeloid cells within tumor stroma, which in turn produces chemokines able to enhance cancer cell survival. Thus, CXCR2 blockers were proposed to improve the efficacy of chemotherapy by blocking this paracrine reaction in the tumor stroma (Acharyya et al., 2012).

Overall this evidence, and the data we provide in this study, make difficult, at least in our tumor model, to match the UC-MSC secretome and the high levels of CXCR2 ligands with the anti-angiogenic and anti-inflammatory properties generally attributed to mesenchymal cells. However, the close cell-to-cell interactions between CSCs and MSCs occurring in spheroids, could completely change their mutual state in the microenvironment, inducing the modulation of adhesion proteins able to inhibit proliferative intracellular pathways; we did not rule out that *in vivo* this mechanism could represent the prevalent condition.

Altogether our results demonstrate that direct (cell-to-cell contact) or indirect (via the release of soluble factors) interactions between GBM CSCs and UC-MSCs in co-culture produce divergent effects on cell growth, invasion and migration, with the former mainly causing an inhibitory response and the latter a stimulatory one, at least in part mediated by the paracrine activation of CXCR2 by its ligands GROs and IL-8, which emerged as main mediators of the indirect activation of cell proliferation.

## Ethics Statement

The study does not involve experimentation on humans but the *in vitro* testing on cells from post-surgical specimens of human glioblastoma, and from umbilical cords. All subjects involved gave written informed consent to provide the relative specimens in accordance with the Declaration of Helsinki. The protocol was approved by the Comitato Etico Azienda Universitaria Ospedaliera San Martino, Genova, Italy.

## Author Contributions

ABa, TF and FB: conceived and designed the experiments; ABa, AP, AC, FB, ABo and MG: performed the experiments; AD, VP and RS: contributed reagents/materials/analysis tools; ABa, TF, FB and AD: wrote the manuscript. All authors analyzed the data.

## Conflict of Interest Statement

The authors declare that the research was conducted in the absence of any commercial or financial relationships that could be construed as a potential conflict of interest.
