# Supplementary material for: Different Effects of Human Umbilical Cord Mesenchymal Stem Cells on Glioblastoma Stem Cells by Direct Cell Interaction or Via Released Soluble Factors
Source: Front Cell Neurosci. 2017 Oct 13;11:312. doi: 10.3389/fncel.2017.00312 (PMC5645520; doi:10.3389/fncel.2017.00312)
Supplement: Supplementary file 1 [file Presentation_1.pdf]

Supplementary Figure 1

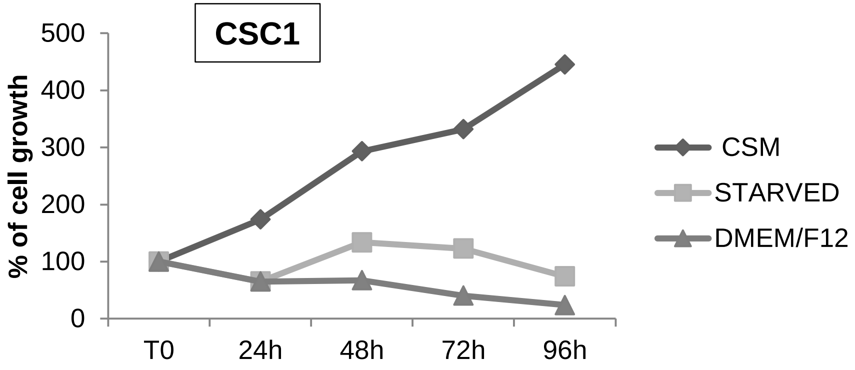

Growth of CSC1 in different media.
CSC1 were plated in complete stem medium on matrigel (T0), grown in complete stem medium (CSM) or in DMEM/F12 (starved DMEM/F12) or plated directly in DMEM/F12 (DMEM/F12). Growth curve was performed using the MTT assay after 24, 48, or 72h.

Supplementary Figure 2

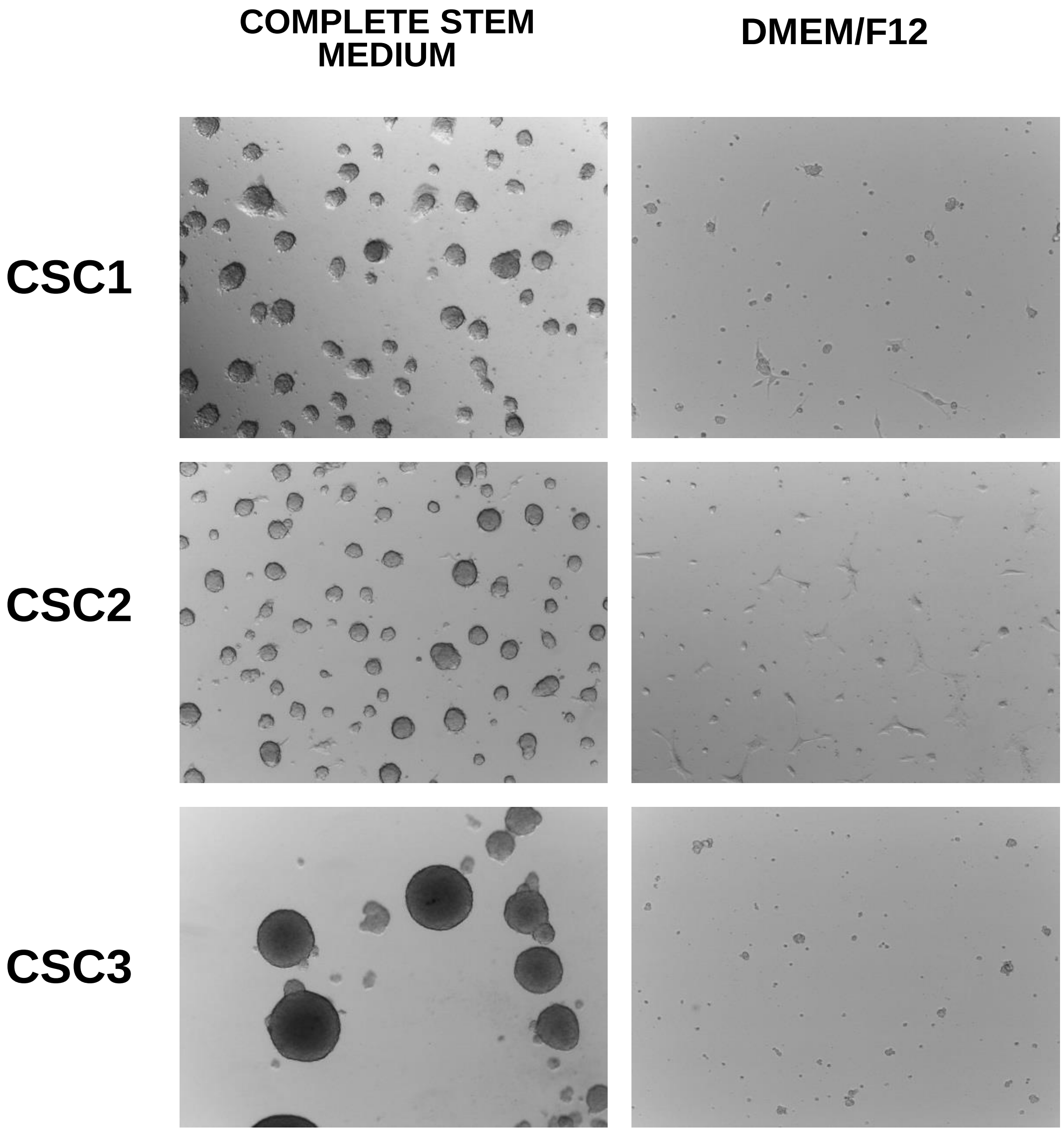

Effects of different media on sphere formation.
Morphological appearance of CSC1, CSC2, CSC3 grown as floating spheres in complete stem medium and as rare adherent non proliferating cells in DMEM/F12 medium.

Supplementary Figure 3

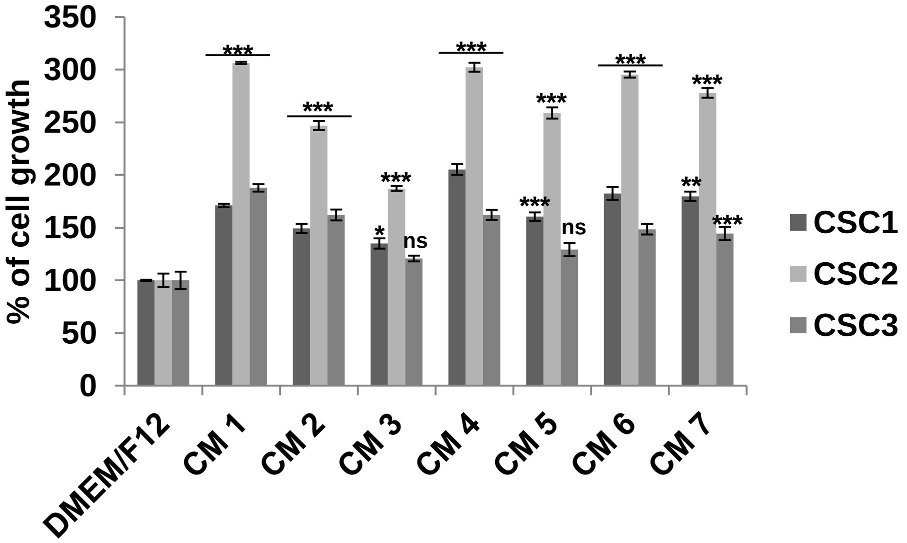

Effects of individual conditioned medium derived from UC-MSCs on CSC1, CSC2, CSC3.
CSC growth was analyzed, after 72h of culture in the absence (DMEM/F12) or presence of UC-MSC-derived conditioned medium (CM) by MTT assay. Statistical analysis was performed with One-way ANOVA $p<0.0001$; with post-hoc analysis by the Dunnett's test. * $p<0.05$; ** $p< 0.01$; *** $p< 0.001$; ns: non statistically significant.

Supplementary Figure 4

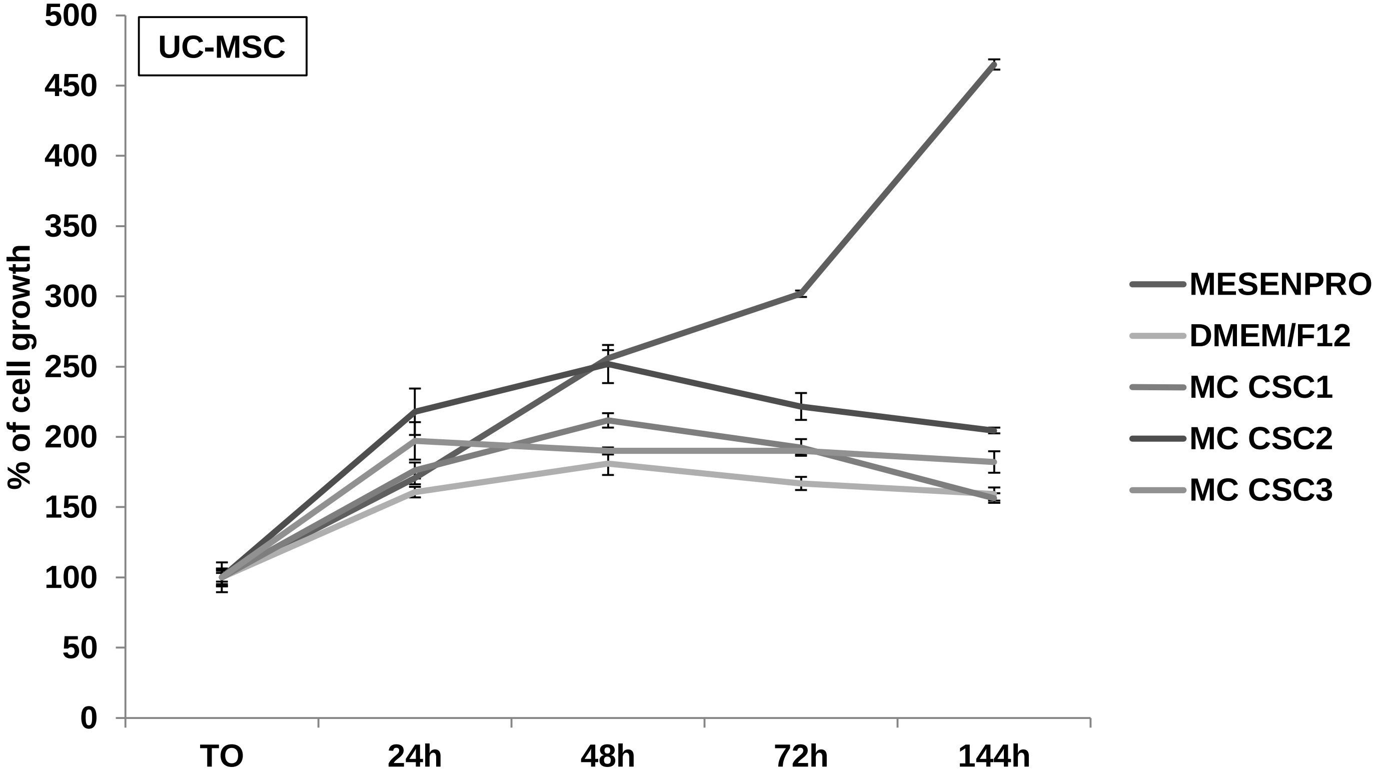

Effects of individual conditioned media derived from CSC1, CSC2, CSC3 on UC-MSC growth.
UC-MSC growth was analyzed by MTT assay in presence of CSCs derived conditioned media (MC) collected from CSC1, CSC2, CSC3 grown in DMEM/F12 for 72h, or DMEM/F12 referred as control. As normal proliferation control, UC-MSC growth was evaluated also in MesenPro medium

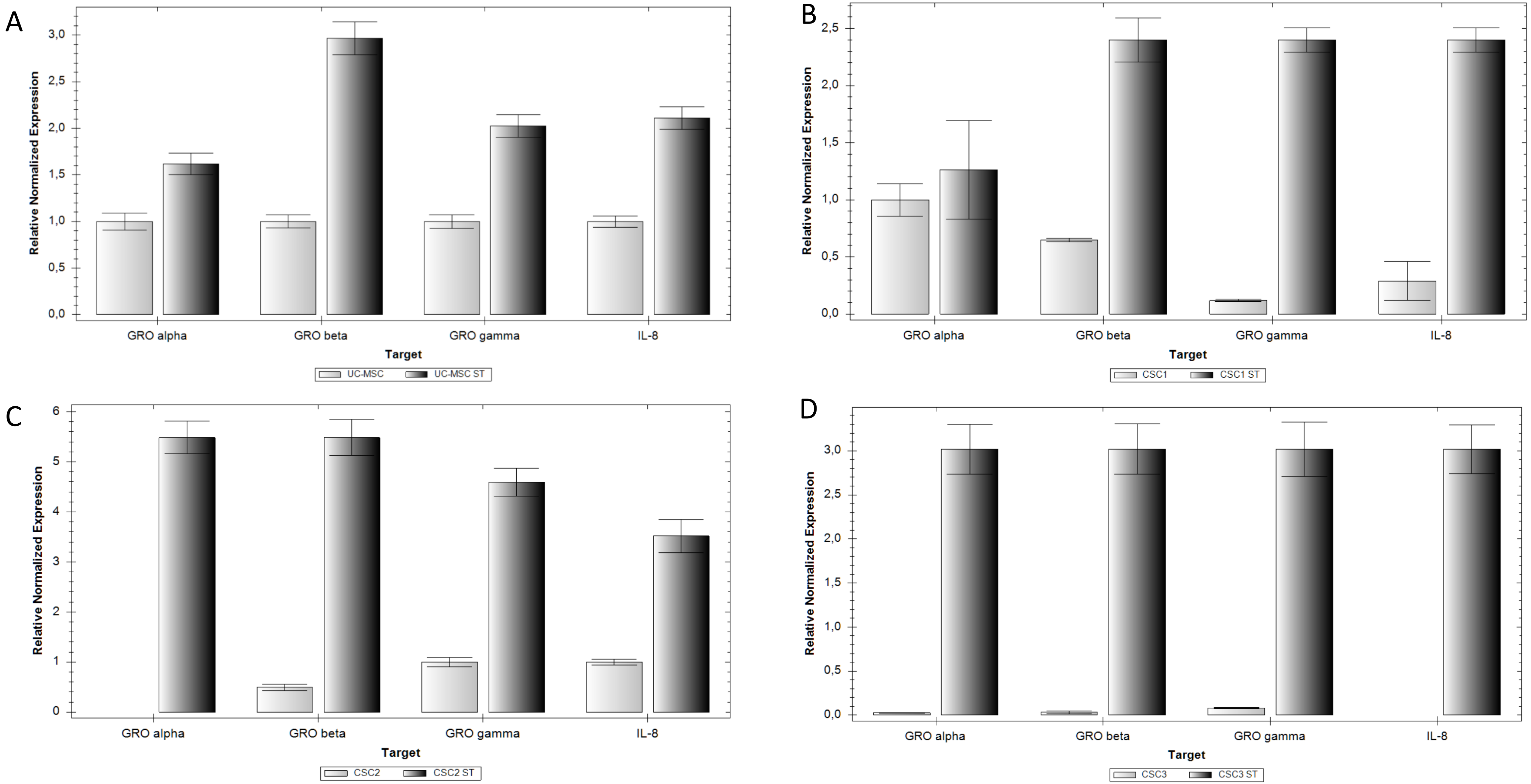

Supplementary Figure 5. qPCR evaluation of GRO alpha, beta and gamma, and IL-8 expression in UC-MSC (A), CSC1 (B), CSC2 (C), and CSC3 (D). Comparison was performed between gene expression in cells grown in complete stem cell medium and serum free medium DMEM/F12 (ST)
